# Single-cell transcriptome and cell type-specific molecular pathways of human non-alcoholic steatohepatitis

**DOI:** 10.1038/s41598-022-16754-7

**Published:** 2022-08-05

**Authors:** Rikard G. Fred, Julie Steen Pedersen, Jonatan J. Thompson, Julie Lee, Pascal N. Timshel, Stefan Stender, Marte Opseth Rygg, Lise Lotte Gluud, Viggo Bjerregaard Kristiansen, Flemming Bendtsen, Torben Hansen, Tune H. Pers

**Affiliations:** 1 Novo Nordisk Foundation Center for Basic Metabolic Research, University of Copenhagen, Copenhagen, Denmark; 2 Gastro Unit, Medical Division, Copenhagen University Hospital Hvidovre, Hvidovre, Denmark; 3 Department of Clinical Biochemistry, Bispebjerg and Frederiksberg Hospital, Copenhagen, Denmark; 4 Gastro Unit, Surgical Division, Copenhagen University Hospital Hvidovre, Hvidovre, Denmark

**Keywords:** Cell biology, Computational biology and bioinformatics, Gastroenterology

## Abstract

The aim of this study is to characterize cell type-specific transcriptional signatures in non-alcoholic steatohepatitis (NASH) to improve our understanding of the disease. We performed single-cell RNA sequencing on liver biopsies from 10 patients with NASH. We applied weighted gene co-expression network analysis and validated our findings using a publicly available RNA sequencing data set derived from 160 patients with non-alcoholic fatty liver disease (NAFLD) and 24 controls with normal liver histology. Our study provides a comprehensive single-cell analysis of NASH pathology in humans, describing 19,627 single-cell transcriptomes from biopsy-proven NASH patients. Our data suggest that the previous notion of ”NASH-associated macrophages” can be explained by an up-regulation of normally existing subpopulations of liver macrophages. Similarly, we describe two distinct populations of activated hepatic stellate cells, associated with the level of fibrosis. Finally, we find that the expression of several circulating markers of NAFLD are co-regulated in hepatocytes together with predicted effector genes from NAFLD genome-wide association studies (GWAS), coupled to abnormalities in the complement system. In sum, our single-cell transcriptomic data set provides insights into novel cell type-specific and general biological processes associated with inflammation and fibrosis, emphasizing the importance of studying cell type-specific biological processes in human NASH.

## Introduction

Non-alcoholic fatty liver disease (NAFLD) is strongly associated with overweight and the metabolic syndrome, affecting approximately 25% of the world’s adult population^1^. Up to 25% of the patients will develop non-alcoholic steatohepatitis (NASH), which may further progress to cirrhosis, hepatocellular carcinoma and liver failure^2–4^. The pathophysiology of NASH involves hepatocyte injury and death. It has lately become clear that fibrosis is the main determinator of development of cirrhosis and mortality in NASH^5^. Currently, there is a lack of understanding of the cellular processes involved in the processes leading to progression from pure fatty liver to NASH and further to cirrhosis. Therefore, it is important to gain more knowledge about the pathophysiological molecular pathways in order to identify biomarkers and to develop targeted preventive and treatment initiatives.

Whereas traditional RNA sequencing (RNA-seq) has been instrumental to characterize genome-wide differences between portal and central hepatocytes, the technique has provided limited insights into the inter-cellular complexity of the liver^6^ especially when it comes to characterizing sub-types of cells; that be different kinds of liver resident macrophages^7^ or rare cell types such as activated hepatic stellate cells, which drive the fibrogenesis^8,9^. The relationship between up- and down-regulation within cell types, or subpopulations, has thus been therefore been inadequately covered by traditional RNA-sequencing studies.

Single-cell RNA sequencing (scRNA-seq) allows for a characterization of tissues at the single-cell resolution by profiling thousands of individual cells. The single-cell transcriptome of the healthy human liver has been described previously^7,10^ and has been followed by studies of cirrhosis in humans^10,11^. So far, NASH have only been studied on the single-cell level in mice models and have yet to be investigated properly in humans^8,12^. To gain a better understanding of the cell type specific disease phenotype in obesity-related NASH, we performed scRNA-seq on liver biopsies from patients with NASH. Furthermore, to robustly detect complex transcriptional signatures associated with NAFLD, we used gene network analysis, which has previously been used to resolve molecular processes and biological pathways related to both NAFLD and NASH^8^.

## Materials and methods

### Study population

We collected liver tissue from 10 morbidly obese patients undergoing bariatric surgery at Copenhagen University Hospital Hvidovre, Denmark. The tissue was sampled as a wedged biopsy from margo inferior of the liver. Sampling was performed immediately after trocar placement and before the actual bariatric procedure. All patients were screened for other aetiologies of liver disease and had no former or ongoing alcohol overuse. Patients fulfilled the requirements set by the Danish Health Authorities for bariatric surgery in Denmark, which among other criteria includes a mandatory pre-surgery weight loss (8% of total body weight)^13^.

### Liver histology and assessment of NASH

A part of the liver biopsy underwent fixation in paraformaldehyde for later paraffin embedment and histological analysis. Histopathological scoring was performed by use of the steatosis-inflammation-fibrosis score (SAF score). All 10 patients had inflammation, ballooning and fibrosis. However, four-out-of-ten patients had a steatosis score of zero while simultaneously having both inflammation, ballooning and fibrosis (Supp. Table 1). This may have been an effect of the required weight loss^14^. Bedossa et al. have previously reported that all patients with a SAF activity score of two or more all had NASH and that the SAF score, although purely morphological, is clinically relevant^15^. Consequently, for this study, we isolated liver cells from 10 patients with a SAF activity score of 2 to study NASH on a single-cell level.”

### Ethics

The study protocol was approved by the the Regional Ethics Commitee in the Capital Region of Denmark (H-17014324) and conducted according to the Declaration of Helsinki. Oral and written informed consent were obtained from all study participants prior to participation.

### Isolation of cells from fresh liver biopsy

Approximately 100 mg of fresh liver tissue was placed in Hypothermosol (Sigma-Aldrich, USA) transport buffer on ice immediately after sampling. The tissue was brought directly to the Novo Nordisk Foundation Center for Basic Metabolic Research for analysis. For dissociation into single cells the tissue was minced and incubated with Liberase low dispase (Sigma-Aldrich, USA) for 20 min at 37 degrees Celsius at 250 rpm. The tissue was triturated by pipetting 10 × with a 1000 µl tip before the enzymatic reaction was stopped by the addition of 2% PBS-FBS and then filtered through a 70 µm cell strainer. Cells were pelleted at 500 rcf for 10 min after which the supernatant was removed and the cells washed once in PBS-BSA. The cells were counted using the Nucleocounter (Chemometec, Denmark).

### Droplet-based single-cell RNA sequencing

The isolated cells were visually inspected and loaded on the Chromium controller (10x Genomics, USA) using the manufacturer’s protocol (Chromium Single Cell 3' Reagent Kits; v2 chemistry) to obtain single-cell libraries that were sequenced on a NextSeq sequencing system (Illumina, USA) using a high yield kit (75 cycles) at 400 million reads per sample. The Cell Ranger 2.0 pipeline (10x  Genomics) was used to align reads, quantify unique molecular identifiers (UMI) and generate filtered feature-barcode expression matrices. The reads were mapped to the hg19 human reference genome following the steps outlined on the 10x Genomics website^16^. The raw expression matrices produced by the Cell Ranger software were analysed using the Seurat v.3.0.0.9 R package^17^. Clusters were annotated through automated label transfer from the previously published data sets by McParland et al.^7^ and Aizarani et al.^10^ as well as manual annotation (Supp. Fig. 1). Further analyses were performed in R and are described in the supplementary methods with all code used available at Github (https://github.com/perslab/fred-2022). Raw sequence data are not made public due to patient privacy.

### Bulk RNA-seq data processing

The Gerhard et al. ^18^ data set was chosen as a reference for the scRNA-seq data because the samples, similar to the samples in the present study, underwent bariatric surgery prior to liver biopsies and because the samples were profiled using RNA-sequencing, which resembles scRNA-seq data better than microarray-based gene expression profiling. RNA-seq fastq files were downloaded from the European Nucleotide Archive (ENA; PRJNA512027) using the ENA browser tools^18,19^. Sequence quality was assessed using fastqc (version 0.11.5) and MultiQC (version 1.8), and samples with fewer than 200 m sequences were removed^20^. Sequences were aligned to the GRCh38 (Ensembl 93) genome assembly using STAR (version 2.7.3a)^20^, with sjdbOverhang set to 82. Binary Alignment Map (BAM) files were sorted using samtools sort (version 1.9) and reads were counted using htseq-count (version 0.11.2). The raw gene counts were assembled as a matrix and normalised using the DESeq data set FromMatrix, estimateSizeFactors and counts functions from the DESeq2 R package^20^. Several samples (DLDR_0037-46 and DLDR_61-62) were relabelled as fibrotic on advice of the corresponding author responsible for generating the data set and an outlier sample (DLDR_0181) was omitted after visual inspection of principal component plots. The data set comprised a total of 160 obese cases and 24 controls labelled by clinical status. All cases in the control bulk RNA-seq data set have undergone a weight loss intervention required for bariatric surgery.

### Module association with disease condition

Modules of co-expressed genes were identified within each cell type using weighted gene co-expression network analysis (WGCNA)^21^. Module co-expression was validated in the bulk RNA-seq dataset and in a scRNA-seq dataset from healthy donors^7^. Module activity in bulk RNA-seq sample and the single-cell RNA-seq data was computed as a weighted mean of scaled module gene expression. The association of module activity with lobular inflammation and fibrosis was computed using multivariate linear models controlling for body mass index, age and sex. Module biological function was assessed by testing for enrichment of gene sets from the Gene Ontology (GO), KEGG and Reactome databases^22–24^. See the Supplementary Materials and Methods for additional detail.

### Mapping of candidate genes for genome-wide association with NAFLD to modules

Two hundred fifteen potentially relevant genes for NAFLD (diagnosed using elevated ALT as a proxy for NAFLD) recently presented by Vujkovic et al.^25^, were mapped to our modules using hypergeometric enrichment tests. The genes are the predicted effector genes for 77 genome-wide loci significantly associated with NAFLD. In brief, the candidate genes were based on DEPICT gene prediction, coding variant linkage analysis, expression quantitative traits locus analysis, and annotation enrichment, and protein–protein interaction networks.

## Results

### Single-cell analysis of human liver from obese patients with NASH

**Figure 1:**
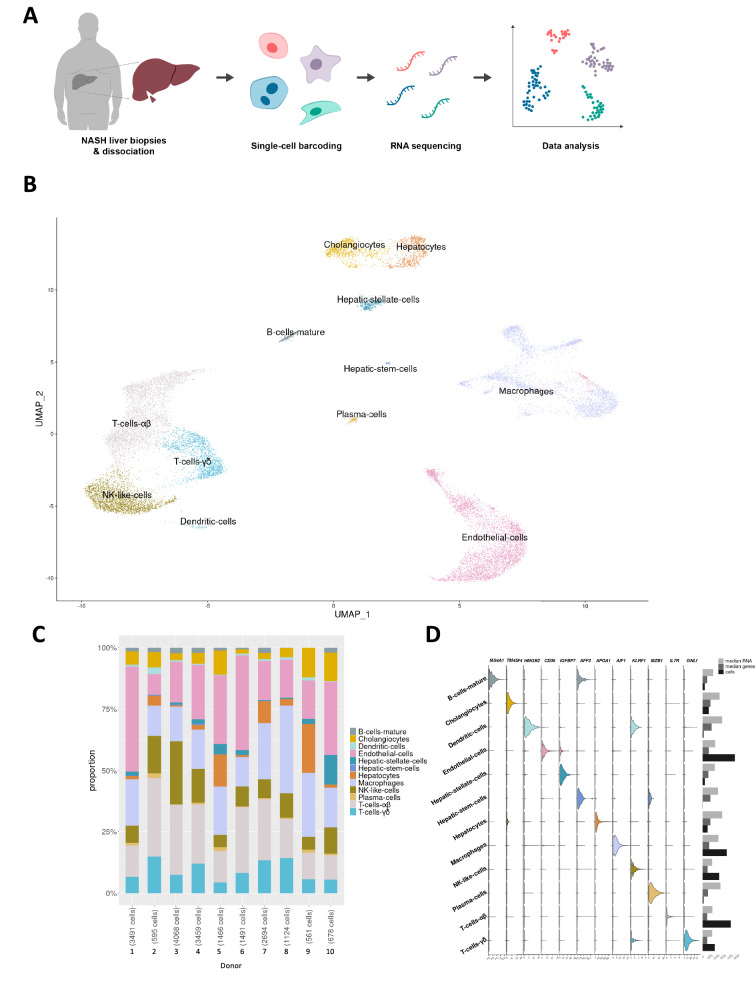
Single-cell analysis of human liver from obese patients with NASH. (**A**) Schematic diagram of the single-cell analysis workflow and analyses. Wedge biopsies from obese patients with NASH were dissociated enzymatically followed by single-cell barcoding, sequencing and data analysis. (**B**) UMAP projection of 19,627 cells showing the assigned identity for the main cell types in the liver, annotated by transfer of labels from previously published data sets. (**C**) Bar graph showing the proportion of cell types in each donor sample. (**D**) Left: Violin plots showing expression of selected marker genes for 12 distinct coarsely clustering cell types. The expression values are normalised, corrected for sequencing confounders and batch corrected for differences in average and variance between donor samples. Right; median number of RNA molecules, median number of genes and total number of cells in each cluster.

To investigate human NASH on a cellular level we performed scRNA-seq analysis of liver biopsy-derived cell populations originating from 10 patients undergoing bariatric surgery (baseline characteristics of the 10 patients are shown in Supp. Table 1; Fig. 1A). Average age and body mass index at the time of surgery was 44 ± 7.5 years and body mass index 43.4 ± 7.4 kg/m^2^, respectively, with an even distribution between sexes (5 females/5 males).

The dataset comprised of 19,627 quality-controlled cells with an average of 1,962 cells (range 561 to 4,068) from each patient. Cells had a median number of 799 detected genes (range 697 to 1,391) and 1,687 unique transcripts (range 1,458 to 2,952). Clustering revealed 20 populations of cells comprising 12 distinct cell types (Fig. 1B). The cell types were parenchymal (hepatocytes), non-parenchymal (cholangiocytes, endothelial cells, macrophages, hepatic stellate cells and hepatic stem cells) and immune cells (B-cells, dendritic cells, NK-like cells, plasma cells and T-cells). Cell types were not patient-specific, with all but two clusters comprising cells from all 10 donors and the remainder composed of cells from at least eight patients (Fig. 1C). Canonical cell type marker genes were used to confirm cluster labels (Fig. 1D). Additional markers can be found in Supp. Fig S1B.

### Multiple cell type-specific transcriptional signatures associate with inflammation and fibrosis

**Figure 2:**
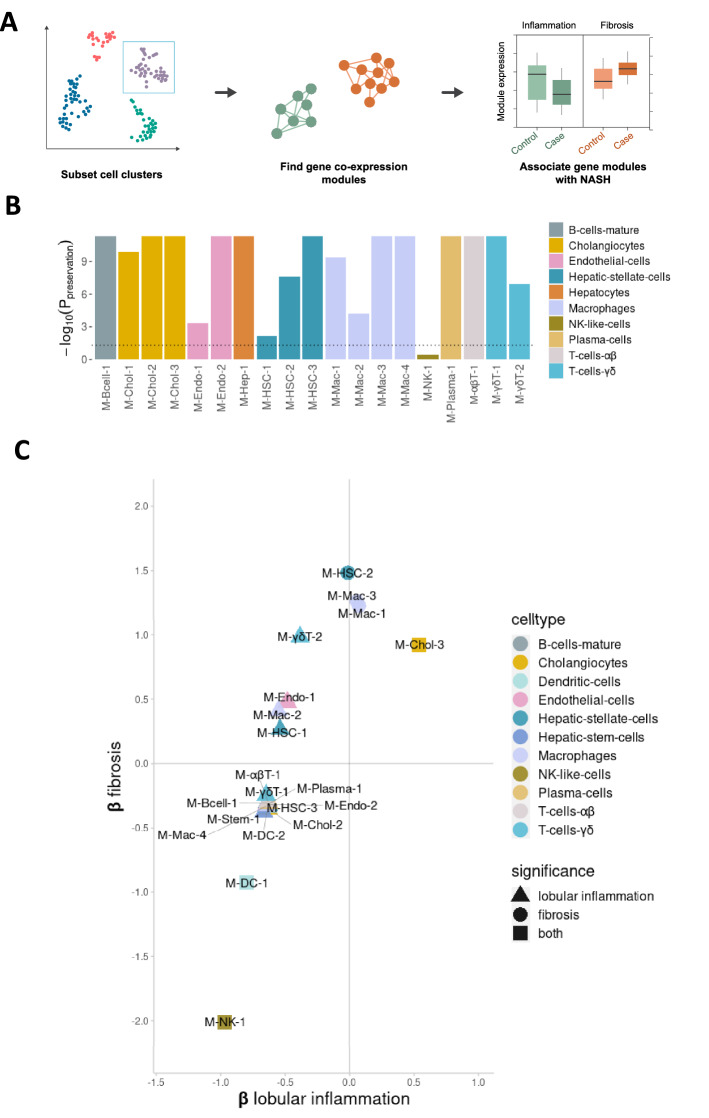
Expression of co-expressed gene modules is associated with both inflammation and fibrosis in NASH. (**A**) Weighted gene co-expression network analysis workflow used to identify disease-associated gene modules. For each of the 12 major cell types, co-expression gene modules were identified. After quality control, 22 high-confidence modules were tested for association between module activity and levels of fibrosis, lobular inflammation and steatosis using a published RNA-seq data set with 24 healthy controls and 160 fatty liver disease samples. (**B**) Bar plot showing strength of module co-expression preservation in the MacParland et al. scRNA-seq liver data set. *P*-values were corrected for multiple testing (Benjamini-Hochberg) and the dotted line marks the significance threshold (*P* < 0.05). Modules are coloured by the cell cluster in which they were identified. Modules from Dendritic cells and Hepatic stem cells, which were absent in the MacParland et al. data set, were omitted in this step. (**C**) Cell type-specific gene modules whose activity pattern is associated, either positively or negatively, with the level of inflammation and fibrosis in fatty liver disease, in multivariate linear regression models with module activity as the outcome variable. The vertical and horizontal axes correspond to standard deviation differences in module activity for each unit change in lobular inflammation (encoded as 0, 1 or 2) or fibrosis (0 or 1). The plot includes only modules with statistically significant association to at least one condition using bootstrap percentile *P*-values corrected for multiple testing within each phenotype (Benjamini-Hochberg). Modules are coloured by cell cluster.

To get further insight into putative biological pathways transcriptionally associated with NASH, we identified sets of co-expressed genes (henceforth ‘modules’) within each major cell type and associated the modules with NASH risk phenotypes (Fig. 2A). Robust weighted gene co-expression network analysis (rWGCNA) yielded a total 22 high-confidence modules across all cell-types (Supp. Table 2). To test whether any of the gene modules detected in the NASH patients could also be identified in healthy liver samples, we tested whether module co-expression was preserved within the corresponding cell types from healthy livers in an existing non-case liver single-cell RNA-seq data set^7^. Eighteen out of 19 modules tested (omitting modules from dendritic cells and hepatic stem cells, which were not present in the external data set) were found to be co-regulated in these healthy liver samples (Fig. 2B), validating the modules and showing them to be present also in the healthy state.

We next assessed whether the modules identified in the scRNA-seq data set were associated with hepatic steatosis, inflammation and/or fibrosis within a large existing bulk RNA-seq data set comprising 24 individuals with normal histology and 160 patients with NAFLD^18^. For each cell population-specific module, we computed the activity in each sample from the external dataset (the summed expressed of all module genes; see Materials and Methods). Linear regression was then used to identify modules whose activity was associated with steatosis, inflammation or fibrosis (*P* < 0.05, corrected for multiple testing, *n* = 22). Whereas none of our modules were significantly associated with steatosis in the external dataset, 17 modules were significantly associated with inflammation and 6 modules with fibrosis (Fig. 2C). Moreover, three modules, M-Chol-3, M-DC-1 and M-NK-1 were associated with both inflammation and fibrosis (Fig. 2C, Supp. Table 3).

Next, in order to link disease-associated modules with putative genetic predisposition, we mapped the 214 predicted effector gene(s) for 77 genome-wide significant genetic loci associated with ALT levels from a recent genome-wide association study (GWAS) for NAFLD^25^ to our modules. Three modules significantly enriched for genes associated with significant loci for ALT levels, namely the hepatocyte module M-Hep-1 (*P* = 1.65 × 10^–7^; overlap *APOC1, APOH, HP, SERPINA1, MLXIPL, MTTP, HSD17B13, SLC2A2, CPS1*), the cholangiocytes module M-Chol-1 (*P* = 5.05 × 10^–5^; overlap *CEBPA, SERPINA1, CCDC18*), and the macrophage module M-Mac-1 (*P* = 5.05e-5; overlap *HLA-DRA, HLA-DQA2, HLA-DQB2*) (Supp. Fig. 2). Although the majority of GWAS loci were only associated with ALT levels, three loci (*APOCI, MTTP*, *APOH*) were associated with both ALT levels and hepatic fat. Of the previously reported risk genes for NAFLD^26^, we found that *HSD17B13* was also part of M-Hep-1 (Supp. Table 2). These findings indicate that the SNPs associated with NAFLD are directly affecting biological processes coupled to inflammation and fibrosis in NASH. They further suggest that a genetic predisposition towards NAFLD affects more cell types than just hepatocytes, in this case cholangiocytes and macrophages.

### Cell type-specific transcriptional signatures indicate a general down-regulation of protein synthesis and oxidative phosphorylation

**Figure 3:**
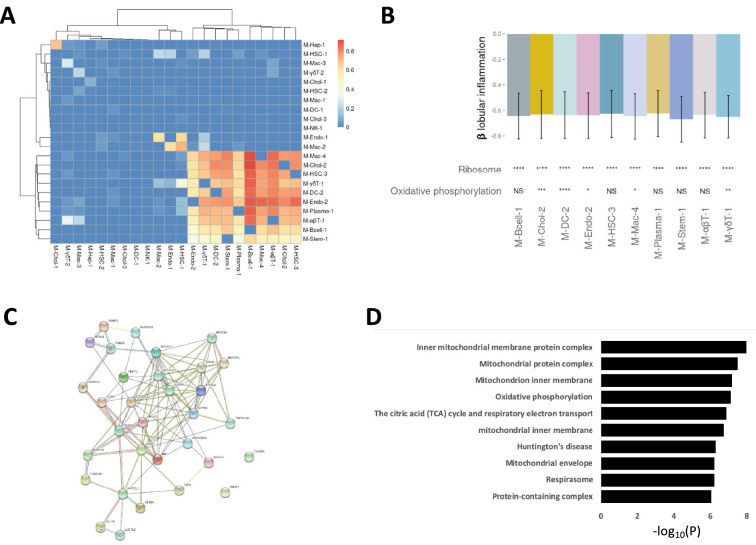
Pervasive down-regulation of genes related to ribosomal translation in inflammation. (**A**) Heatmap showing the proportion of genes in a module (columns) shared with other modules (rows). The overlap of a cluster with itself was set to 0 and columns and rows were clustered hierarchically. (B) Top; bar plot shows standard deviation differences in module activity for each unit change in lobular inflammation (encoded as 0, 1 or 2) in a multivariate linear regression model with module activity as the outcome variable. Error bars show 0.95 confidence intervals, obtained using bootstrap percentiles with bootstrap replicates. Bottom; module enrichment for three top-shared KEGG terms identified using GERR. (**C**) Protein–protein interaction network of the 32 overlapping genes correlated with oxidative phosphorylation. (**D**) Bar plot showing the top ten associated gene sets from Gene Ontology, based on the 32 overlapping genes correlated with oxidative phosphorylation. Significance notation refers to P-values obtained using a hypergeometric test and correcting for multiple testing (Benjamini-Hochberg, n = 30). ns: *P* > 0.05; *: *P* ≤ 0.05; **: *P* ≤ 0.01; ***: *P* ≤ 0.001; ****: *P* ≤ 0.0001.

We found that 10 modules expressed across multiple cell types comprised partially overlapping genes (lower right corner in Fig. 3A). Gene set analysis of these modules (see Methods and Materials) revealed a general down-regulation of protein synthesis and a partial down-regulation of oxidative phosphorylation in association with inflammation (Fig. 3B). To further investigate this finding, we took the four modules (M-Chol-2, M-Endo-1, M-HSC-1, M-Mac-2) that correlated with oxidative phosphorylation and identified all genes that where part of at least three of the four modules. Using the STRING database we next identified protein–protein partners for the 32 gene products. The protein–protein interaction network comprised a highly connected network of protein–protein interactions with a total of 102 interactions (*p*-value < 2.22 × 10^−16^) (Fig. 3C). Based on these specific proteins we identified the underlying biological processes, using the GO database (Fig. 3D, Supp. Table 8). The enriched GO terms expectedly pointed to processes related to mitochondrial respiration, the most enriched GO term being “inner mitochondrial membrane protein complex” (*p*-value < 1.04 × 10^−8^). An analysis of the descendent terms implicated the more specific GO term “mitochondrial respiratory chain complex I” (*p*-value < 8.60 × 10^−4^), indicating that the general down-regulation of oxidative phosphorylation is mainly in complex I. Interestingly, except hepatocytes and NK-cells, all 10 remaining cell types had at least one module with overlapping genes, including the remaining immune cell populations. This commonality suggests that there is a drive towards down-regulation of protein synthesis in cell populations that are typically activated during inflammation. This down-regulation of ribosomal activity associated to inflammation could also be seen in M-HSC-1 even though this module did not contain the same set of genes as the previously mentioned modules, indicating that ribosomal activity and mitochondrial function is regulated via several sets of genes in hepatic stellate cells. In dendritic cells, M-DC-1 exhibited down-regulation of genes associated with cell division and in cholangiocytes, M-Chol-3 indicated an up-regulation in MAPK kinase activity in both inflammation and fibrosis and M-gdT-2 shows an alteration in T-cell receptor signalling in response to inflammation. The only module that was not co-expressed in normal liver (see Fig. 2C; Suppl. Table 8) was M-NK-1. This module is negatively associated with both inflammation and fibrosis. Gene set analysis showed that the module was associated with the dysregulation of three different transporters, *SLC26A3, SLC27A4* and *SLC12A3* (Suppl. Table 6). These results indicate that mitochondrial dysfunction associated with NAFLD is specific for a subset of cell-types while downregulation of ribosomal activity is synchronized between the majority of cell types in the liver, although via cell-type specific pathways.

### Calgranulin expressing, fibrosis-associated macrophages constitute a novel sub-population present in normal liver and upregulated in NASH

**Figure 4:**
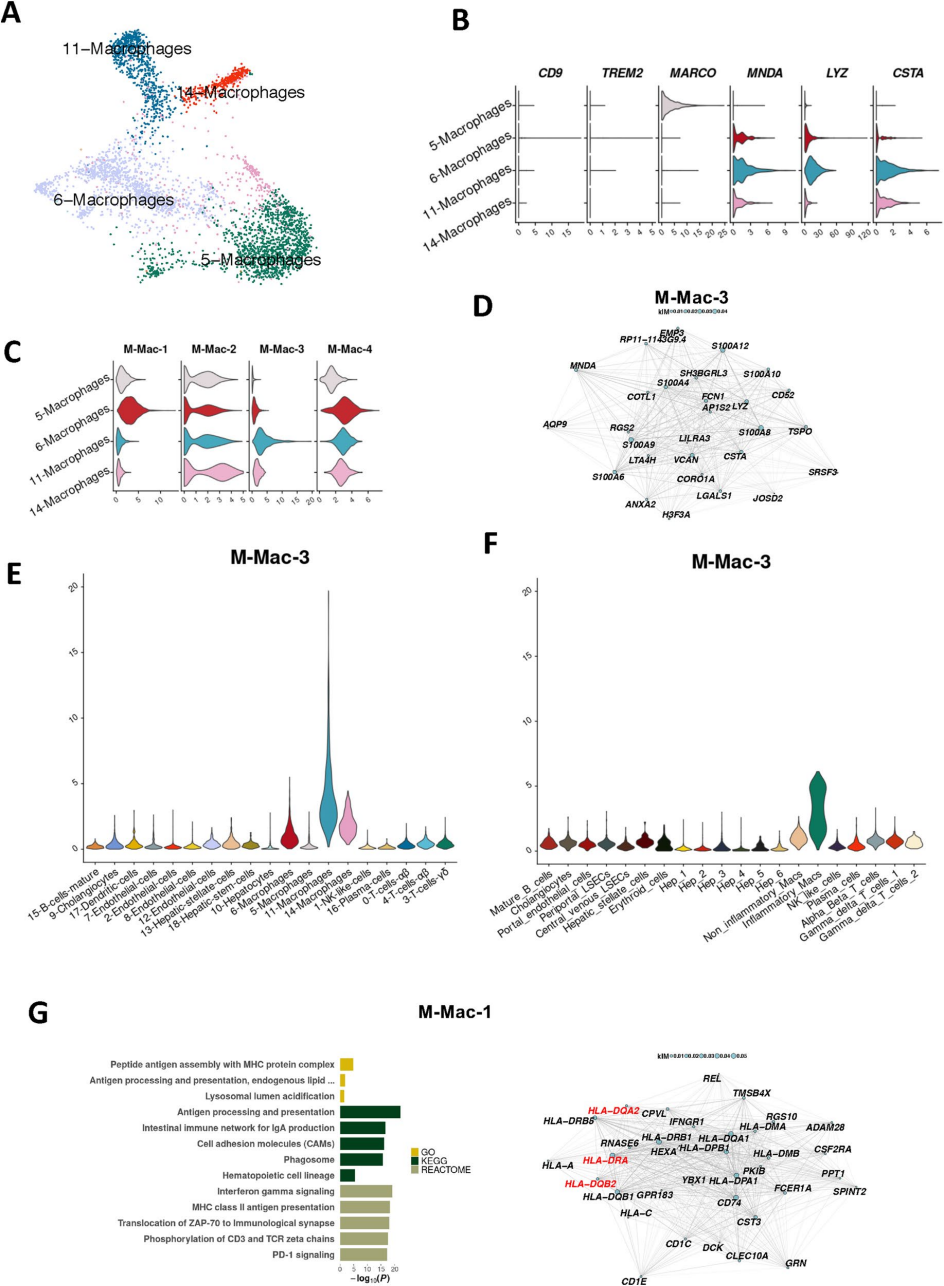
A subset of pro-fibrotic macrophages associates with the M-Mac-3 module. (**A**) UMAP projection of the four macrophage clusters. (**B**) Violin plots of normalised and batch-corrected expression of macrophage specific marker genes. (**C**) Violin plots of the activity of macrophage modules, where some modules appear to be cluster-specific. (**D**) M-Mac-3, of which the 30 most central genes are seen in the network diagram. (E–F) Normalised and batch corrected gene expression of M-Mac-3 in the single-cell RNA-seq samples from individuals with NASH (**E**) and normalised (but not batch corrected) gene expression in normal liver samples from the MacParland et al. data set (**F**). (**G**) M-Mac-1, of which the 30 most central genes are seen in the network diagram (right panel), enriched for processes related to antigen presentation (left panel). In the network diagrams, node sizes represent gene centrality in the module (‘kIM’), and edge widths represent the strength of gene–gene co-expression. For the bar plot, gene sets from the Gene Ontology (GO), Kyoto Encyclopaedia of Genes and Genomes (KEGG) and Reactome databases were queried. Only significant results were plotted (*P* < 0.05).

Previous studies have implicated liver macrophages in fatty liver disease^12,27,28^. To assess whether liver disease-associated modules were expressed in macrophage subtypes, we separately analysed the four macrophage cell populations from our initial clustering comprising 3,613 macrophage cells that passed quality control (Fig. 4A). Markers for liver macrophage subtypes showed that macrophage cluster 5 exclusively expressed the gene for macrophage receptor with collagenous structure (*MARCO*), designating these macrophages as Kuppfer cells. Macrophage clusters 6, 11 and 14 expressed myeloid cell nuclear differentiation antigen (*MNDA*), showing that these cells were derived from circulating monocytes. These clusters also expressed the pro-inflammatory markers Lysozyme C (*LYZ*) and Cystatin A (*CSTA*) (Fig. 4B). Previous findings have associated a subset of *TREM2*^+^*CD9*^+^ macrophages as scar-associated macrophages in cirrhotic liver^12^. We observed a very low number of these activated macrophages in our data set; only 1% of the total macrophages (41/3,613 cells) were *TREM2*^+^*CD9*^+^ (Fig. 4B), suggesting that the population of *TREM2*^+^*CD9*^+^ macrophages potentially are less prevalent in human NASH compared to mouse NASH models^12^. Next, we mapped the macrophage-derived modules to the four macrophage subclusters to further specify their likely location. We found that macrophage cluster 11, a subset of monocyte derived pro-inflammatory macrophages, specifically expressed the M-Mac-3 module genes and that macrophage cluster 6 specifically expressed the M-Mac-1 module (Fig. 4C). Both these modules were significantly associated with fibrosis (Suppl. Table 3). The top genes of the M-Mac-3 module included calgranulin genes (*S100A8/A9*) and *Versican* (*VCAN*) (Fig. 4D). Calgranulin has been implicated in NAFLD/NASH previously^28^ but these new findings indicate that the expression in human liver mainly comes from a small sub-population of macrophages. *VCAN* has likewise been implicated in NAFLD/NASH previously but has not been known to be exclusively expressed in macrophages.

To identify modules specific to macrophage subclusters, we plotted the module gene activity levels in every cell cluster in our data set (Fig. 4E) and in the healthy liver data set (Fig. 4F). M-Mac-3 was specific to inflammatory macrophages in both data sets with considerably lower expression levels in the normal liver samples making up the MacParland et al. data set. We next examined M-Mac-1, the module that also was significantly enriched for the GWAS-derived candidate effector genes associated with ALT levels, *HLA-DRA, HLA-DQA2* and *HLA-DQB2*. These genes were also top genes in the module (median 60^th^ percentile among all module genes; Fig. 4G). Gene set analysis returned several pathways related to antigen presentation and the histocompatibility complex class II protein complex, which includes the aforementioned genes (Suppl. Table 2). These results indicate that the macrophage subpopulations found in NASH are present in healthy tissue as well; rather than NASH driving the formation of disease-specific macrophages, our results suggest than an already existing subpopulation of macrophages increases its level of gene expression in NASH.

### A *RBP1*^+^ subpopulation of activated hepatic stellate cells express collagen in a fibrosis associated manner

**Figure 5:**
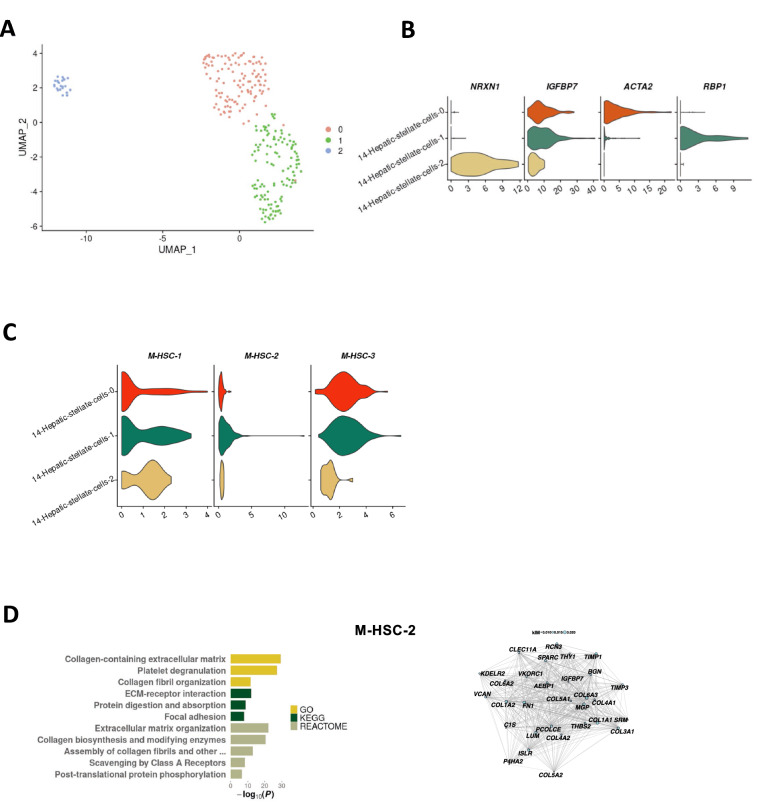
Activated human hepatic stellate cells are divided in two functionally specific subpopulations. (**A**) UMAP projection of the three hepatic stellate cell clusters. (**B**) Violin plots of normalised and batch-corrected expression of hepatic stellate cell marker genes. (**C**) Violin plots of the activity of hepatic stellate cell modules. (**D**) M-HSC-2, of which the 30 most central genes are seen in the network diagram. In the network diagram, node sizes represent gene centrality in the module (‘kIM’), and edge widths represent the strength of gene–gene co-expression. For the bar plot, gene sets from the Gene Ontology (GO), Kyoto Encyclopaedia of Genes and Genomes (KEGG) and Reactome databases were queried. Only significant results were plotted (*P* < 0.05).

Subsetting and re-clustering the hepatic stellate cells revealed three major subpopulations (Fig. 5A). The three populations expressed the general stellate cell marker gene *IGFBP7*. One population expressed *NRXN1,* indicating that this population consists of quiescent stellate cells ^29^. The activated hepatic stellate cells were clearly divided in two sub-populations, being either *ACTA2*^+^ or *RBP1*^+^ (Fig. 5B). When mapping the hepatic stellate cell modules to the different subpopulations, we found that M-HSC-2 mapped exclusively to the *RBP1*^+^ activated stellate cells (Fig. 5C). The M-HSC-2 module includes six collagen genes: *COL1A1*, *COL5A2*, *COL4A1*, *COL3A1*, *COL1A2*, *COL4A2* and the two matrix remodeling genes *TIMP1* and *TIMP3* (Fig. 5D). Further examination using the GO database revealed that M-HSC-2 is highly associated with extra cellular matrix formation and organization (Fig. 5D) predicting that the formation of fibrosis in NASH is due to a *RBP1*^+^ subpopulation of the activated stellate cells.

### Circulating markers for NAFLD are linked to abnormalities in the complement system

**Figure 6:**
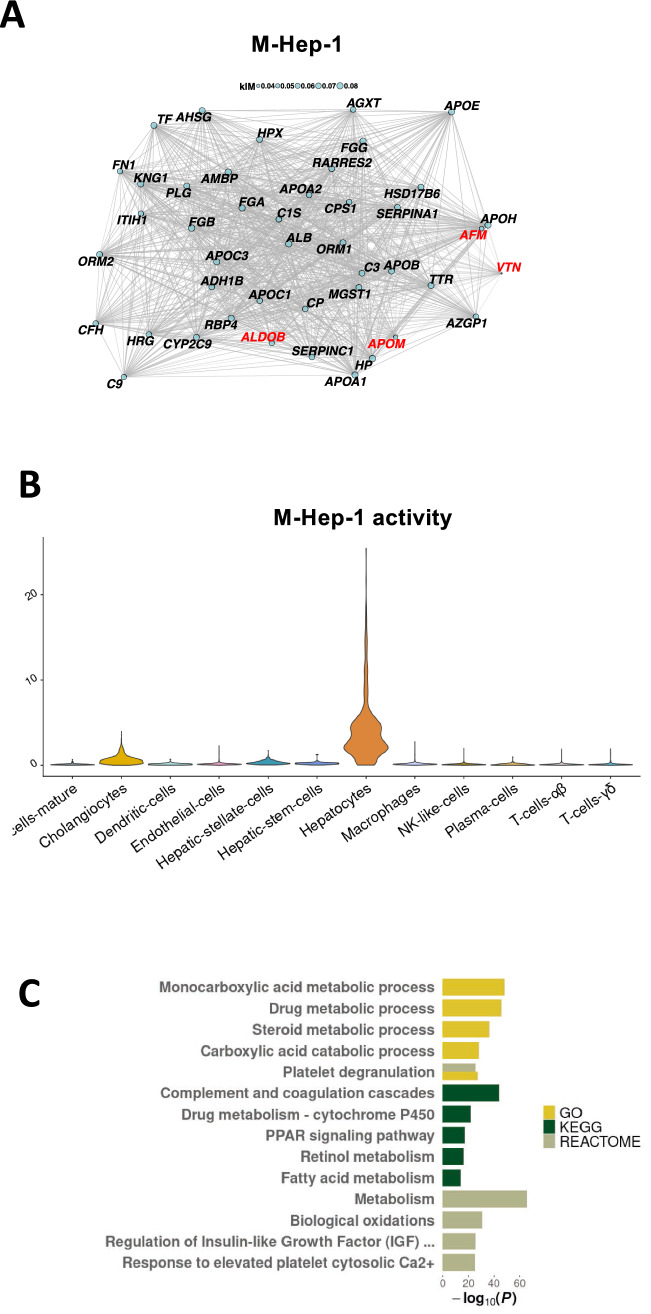
WGCNA reveals that multiple circulating markers of NAFLD are co-regulated. (**A**) Network plot showing the 30 most central genes of M-Hep-8, as well as *ALDOB*, *APOM*, *VTN*, and *AFM*, four out of six recently identified NAFLD plasma proteome markers. Edge width is proportional to gene–gene co-expression and node size is proportional to gene ‘kIM’ centrality (Methods). (**B**) Violin plot showing the activity of M-Hep-1 in 12 cell clusters. (**C**) The biological function of module M-Hep-1, identified through gene set analysis using the Gene Ontology, Kyoto Encyclopedia of Genes and Genomes and Reactome databases. Up to five of the top hits from each database shown. Only significant results were plotted (*P* < 0.05).

Niu et al.^30^ recently identified associations between NAFLD and changes in the plasma proteome levels of six proteins, namely ALDOB, APOM, LGALS3BP, PIGR, VTN and AFM, constituting promising markers for diagnosis of patients with NAFLD. In order to better understand their cellular origins, we tested if these proteins where part of any modules in our scRNA-seq data set. Interestingly, five of these markers (*ALDOB*, *APOM*, *LGALS3BP*, *VTN*, *AFM*) were expressed in hepatocytes, and four (*ALDOB*, *APOM*, *VTN*, *AFM*) were part of the M-Hep-1 module (Fig. 6A). This module was also enriched for the candidate effector genes based on the NAFLD GWAS (Suppl. Fig. 2). An examination of the M-Hep-1 module, which was highly specific to the hepatocyte cell cluster (Fig. 6B), showed that it also included another known marker of NAFLD released from hepatocytes, namely the enzyme betaine-homocysteine s-methyltransferase (*BHMT)*^31^. Gene set tests against GO, KEGG and Reactome showed that the M-Hep-1 module enriched for processes in the complement system, which has previously been linked to fatty liver disease^32^ (Fig. 6C). This is further supported by the presence of vitronectin (*VTN*), a terminal pathway inhibitor^33^. The included markers also associated with both *HNF-1* and *HNF-3α*, transcription factors which have previously been shown to regulate the complement system^34^ and therefore might be the source of the observed changes in the plasma proteome during fatty liver disease. Finally, three of the predicted ALT GWAS genes that are co-regulated in M-Hep-1 (*APOC1, HP, SERPINA1)* are all implicated in complement system activation^33,35^. In sum, these findings indicate that the NAFLD associated loci affects the complement system in the hepatocytes. NAFLD instigates changes in the expression of the co-regulated genes comprising M-Hep-1, which can then be detected in the circulation.

Finally, we found that polymeric immunoglobulin receptor (*PIGR*) was the only circulating biomarker-encoding gene expressed in cholangiocytes in NASH (Suppl. Fig. 4), which would further implicate the cholangiocytes in early changes in fatty liver disease. It is possible that the elevated circulating levels of PIGR stem from early cholangiocyte lipoapotosis, as previously reported^36^. In summary, we find that verified circulating markers of NAFLD are co-expressed in the hepatocytes and that the process seems to be regulated by disturbances in the complement system that are coupled to a genetic pre-disposition.

## Discussion

To provide further insight into the cellular processes of NASH, we here provide a single-cell resolution transcriptomic map of the human NASH liver. Our data set of 19,627 cells from 10 patients with biopsy-proven NASH includes all cell types present in the liver and represents an important resource for future research. We identified gene co-expression modules at the single-cell level and leveraged an existing dataset comprising 184 bulk RNA-seq samples to validate and associate modules with inflammation and fibrosis. The analysis yielded a set of findings confirming and extending existing knowledge about NASH: (1) the general down-regulation of ribosomal activity and mitochondrial dysfunction in NASH occurs across several cell types including the immune cells; (2) human NASH involves four subpopulations of macrophages with distinct expression signatures; (3) apart from the quiescent stellate cells there are two distinct populations of activated human hepatic stellate cells; and (4) multiple circulating markers for NAFLD are co-expressed in hepatocytes and are connected to complement dysfunction of which *PIGR* is predominantly expressed in cholangiocytes.

A key finding was the general down-regulation in ribosomal activity and oxidative phosphorylation associated with inflammation in NASH patients across most cell types in the liver. These general effects of NASH have been shown before but not to the extent that we find here, where practically all cell types in the liver are affected. These results indicate that the liver dysfunction seen in NASH affects the majority of cell types. Protein interaction network of the genes involved in oxidative phosphorylation highlighted the role of the mitochondrial respiratory chain complex I, including the ubiquinone proteins (NDUFC2, NDUFA11/13 and SDHD). This finding provides specific mechanisms for the previously reported reduction of oxidative phosphorylation in NASH^37^.

Another key finding was that macrophage populations are differentially activated in NASH. In particular, calgranulin genes (*S100A8/A9/A12*) and versican, a known modulator of hepatic fibrosis, were almost exclusively expressed in one subpopulation of the three inflammatory macrophage cell populations. The transcriptional module M-Mac-3 was positively associated with fibrosis and present in normal liver samples, but at significantly lower levels and up-regulated in association with fibrosis. Interestingly, this up-regulation of calprotectin (*S100A8/A9*) is the opposite of the observed down-regulation of calprotectin in mice during obesity-related NASH, indicating a potential species-specific divergence. Moreover, we identified an increased expression of versican in macrophages, which also point to a more active role of this subpopulation of macrophages in the modulation of extracellular matrix during fibrosis, in contrast to the current focus on hepatic stellate cells. Taken together, these findings point towards the fact that there is not an emergence of “NASH-associated macrophages” as much as there is a normal subpopulation of macrophages which is upregulated during NASH. We also found that M-Mac-1 included three genes, *HLA-DRA, HLA-DQA2* and *HLA-DQB2,* associated with NAFLD loci. This emphasizes both the importance of the histocompatibility complex class II protein complex in the inflammatory process of NAFLD/NASH as well as the importance of further investigations of the subpopulations of inflammatory liver macrophages.

Re-clustering of the hepatic stellate cells showed three stellate cell populations of which two populations were activated. This is in contrast to a previous paper postulating two populations^38^. If this finding is NASH-specific remains to be seen. The majority (91%) of hepatic stellate cells were activated and the two specific subclusters of activated cells were characterized by the expression of *ACTA2* and *RBP1*. The expression of *ACTA2* identifies this population as myofibroblasts and has been implicated in wound healing specifically^39^. The second population of activated hepatic stellate cells express *RBP1*, which has been implicated in hepatic stellate cell hypertrophy^38^. Interestingly, we also found that a gene module, M-HSC-2, was almost exclusively expressed by the *RBP1*^+^cluster. M-HSC-2 is associated with fibrosis and contains several collagen links. This finding implicates that only a subpopulation of the activated hepatic stellate cells contributes to fibrosis in NASH by collagen production, warranting further investigation.

In the present study, we also found that five circulating markers of NAFLD; *ALDOB*, *APOM*, *VTN*, *AFM* and *BHMT*, were co-expressed and likely regulated by disturbances in the complement system. The module M-Hep-1, which is present in normal liver tissue and related to metabolic pathways, also include the predicted effector genes (*APOC1, HP, SERPINA1)* from NAFLD GWAS that are complement-related components. This finding provides a potential link between genetic pre-disposition to NAFLD, where the associated loci affects the complement signalling in the hepatocytes, and the dysregulation of the co-expressed genes leads to the release of circulating markers. The expression of *PIGR* in cholangiocytes also confirms previous suggestions that secretory component-mediated transfer of IgA is only carried out by intrahepatic biliary epithelium, and not hepatocytes, in human liver^40^.

Despite its strengths, our work comes with a number of limitations. First, the relatively small number (*n* = 10) of patients analysed at the single-cell level and a lack of healthy controls restricted the ability to associate modules with NASH using the single-cell data alone. Thus, the findings reported throughout the paper are based on single-cell data from cases only and hence further studies are needed to distinguish to what degree our findings can be traced back to changes in normal tissue. Drawing on a larger published bulk RNA sequencing data (*n* = 184) set enabled us to associate modules to fibrosis and lobular inflammation. The drawbacks of this approach were the exclusion of modules not co-expressed in the bulk data set and the possibility of module activity corresponding to changes in the abundances of certain cell types. Analysis of module activity in the MacParland et al. scRNA-seq data set, composed of five normal liver samples, partly helped to clarify these issues. Nevertheless, it is plausible that a larger cohort would have expanded the scope of our findings and the clinical significance of our results. Additionally, we acknowledge that complementary methods such as bulk RNA-seq or single molecule fluorescence in situ hybridization may have helped to further confirm the single-cell-based results reported throughout this study. Second, the low number of hepatocytes that survived the dissociation makes it difficult for us to do any in depth analysis of, for example, hepatocyte zonation. For these kinds of studies nuclei will most likely serve as a better ground for analysis as more hepatocytes are likely to be generated, however, on the expense of the other cell types. Third, we were only able to detect a small number of *CD9*^+^/*TREM2*^+^ macrophages, indicating that this population is more associated with cirrhosis, as have been shown before, rather than NASH. There is a possibility that there exist more *CD9*^+^/*TREM2*^+^ macrophages then we were able to find but since we analysed 3,613 macrophages that prospect is rather small. Fourth, it would have been of interest to compare the transcriptional signatures of fibrotic and non-fibrotic NASH individuals, however, unfortunately the present study did not include any patients with non-fibrotic NASH. We speculate that lack of non-fibrotic NASH cases is because individuals undergoing bariatric surgery might have a lower degree of non-fibrotic NASH compared to the general population. Finally, we note that there was a slight difference in amount of required weight loss in the Gerhard et al. and our study in that the Danish Health authorities requires an 8% weight loss whereas the bariatric surgery program at the Center for Nutrition and Weight Management at Geisinger Clinic (reference population) requires a 10% pre-surgery weight loss.

In sum, our findings provide the first transcriptional landscape of human NASH at the single-cell level while providing insights into novel cell type specific and general biological processes associated with inflammation and fibrosis in human NASH.

## Supplementary Information

Supplementary Information 1.

Supplementary Information 2.

## Data Availability

Raw sequence data are not made public due to patient privacy.
