# Supplementary material for: Single-cell transcriptome and cell type-specific molecular pathways of human non-alcoholic steatohepatitis
Source: Sci Rep. 2022 Aug 5;12:13484. doi: 10.1038/s41598-022-16754-7 (PMC9355943; doi:10.1038/s41598-022-16754-7)
Supplement: Supplementary file 1 — Supplementary Information 1. [file 41598_2022_16754_MOESM1_ESM.pdf]

Single-cell transcriptome and cell type specific molecular pathways of human nonalcoholic steatohepatitis

Rikard G Fred, Julie Steen Pedersen, Jonatan J Thompson, Julie Lee, Pascal N Timshel, Stefan Stender, Marte Opseth Rygg, Lise Lotte Gluud, Viggo Bjerregaard Kristiansen, Flemming Bendtsen, Torben Hansen, Tune H Pers

Table of Contents

Supplementary methods *2*

Study population 2

Liver histology and assessment of NASH 2

Ethics 2

Isolation of cells from fresh liver biopsy 3

Droplet-based single-cell RNA sequencing 3

Single-cell RNA sequencing read alignment 3

Quality control, analysis and visualization of single-cell expression data 3

Single-cell RNA sequencing cell cluster annotations 4

Bulk RNA-seq data processing 5

Robust Weighted Gene Co-expression Network Analysis 5

Cell type subsetting and gene filtering 5

rWGCNA adjacency and Topological Overlap Matrix computation 6

Post-rWGCNA gene module filtering 6

Module preservation in control single-cell RNA-seq data set 7

Module association with disease condition 7

Functional module annotation through gene set enrichment testing 8

Mapping of candidate genes for genome-wide association with NAFLD to modules 8

Supplementary results *9*

Mapping of bulk modules to single-cell data set 9

Supplementary figures *10*

Supplementary references *13*

Supplementary methods

Study population

We collected liver tissue from 10 morbidly obese patients undergoing bariatric surgery at Copenhagen University Hospital Hvidovre, Denmark. The tissue was sampled as a wedged biopsy from margo inferior of the liver. Sampling was performed immediately after trocar placement and before the actual bariatric procedure. All patients were screened for other aetiologies of liver disease and had no former or ongoing alcohol overuse. Patients fulfilled the requirements set by the Danish Health Authorities for bariatric surgery in Denmark, which among other criteria includes a mandatory pre-surgery weight loss (8% of total body weight) (Dixon et al., 2004).

Liver histology and assessment of NASH

A part of the liver biopsy underwent fixation in paraformaldehyde for later paraffin embedment and histological analysis. Histolpathological scoring was performed by use of the steatosis-inflammation-fibrosis score (SAF score).

All 10 patients had inflammation, ballooning *and* fibrosis. However, four-out-of-ten patients had a steatosis score of zero while simultaneously having both inflammation, ballooning and fibrosis (**Supp. Table 1**). This may have been an effect of the required weight loss (Vilar-Gomez et al., 2015).

Bedossa et al. have previously reported that all patients with a SAF activity score of two or more all had NASH and that the SAF score, although purely morphological, is clinically relevant (Bedossa et al., 2012). Consequently, for this study, we isolated liver cells from 10 patients with a SAF activity score of 2 to study NASH on a single-cell level."

Ethics

The study protocol was approved by the Regional Ethics Commitee in the Capital Region of Denmark (H-17014324) and conducted according to the Declaration of Helsinki. Oral and written informed consent were obtained from all study participants prior to participation.

Isolation of cells from fresh liver biopsy

Approximately 100mg of fresh liver tissue placed in Hypothermosol (Sigma-Aldrich, USA) transport buffer in ice immediately after sampling. was brought directly to the Novo Nordisk Foundation Center for Basic Metabolic Research for analysis. For dissociation into single cells the tissue was minced and incubated with Liberase low dispase (Sigma-Aldrich, USA) for 20 min at 37 degrees Celsius at 250 rpm. The tissue was triturated by pipetting 10x with a 1000 µl tip before the enzymatic reaction was stopped by the addition of 2% PBS-FBS and then filtered through a 70µm cell strainer. Cells were pelleted at 500 rcf for 10 min after which the supernatant was removed and the cells wash once in PBS-BSA. The cells were counted using the Nucleocounter (Chemometec, Denmark).

Droplet-based single-cell RNA sequencing

The isolated cells were visually inspected and loaded on the Chromium controller (10x Genomics, USA) using the manufacturer's protocol (Chromium Single Cell 3' Reagent Kits; v2 chemistry) to obtain single-cell libraries that were sequenced on a NextSeq sequencing system (Illumina, USA) using a high yield kit (75 cycles) at 400 million reads per sample.

Single-cell RNA sequencing read alignment

The Cell Ranger 2.0 pipeline (10x Genomics) was used to align reads, quantify unique molecular identifiers (UMI) and generate filtered feature-barcode expression matrices. The reads were mapped to the hg19 human reference genome following the steps outlined on the 10x Genomics website (10x Genomics).

Quality control, analysis and visualization of single-cell expression data

The raw expression matrices produced by the Cell Ranger software were analysed using the Seurat v.3.0.0.9 R package (Picelli, 2017). In line with 10x Genomics indications (Picelli, 2017), for each sample, based on an estimated 9,000 cells loaded, the 5,157 barcodes with the highest number of counts were initially selected for downstream analysis. In a further filtering step, based on visual inspection of quality control plots, barcodes with at least 1,000 UMI and proportions of mitochondrial and ribosomal RNA below 0.4 and 0.3, respectively, were retained. The DoubletFinder v3 R package (Picelli, 2017) was used to remove suspected doublets. Samples were normalised individually using the SCTransform() command, which was also used to regress out

confounding variation associated with UMI count, the percentage of mitochondrial gene UMIs, and cell cycle, following the steps in ref. (Picelli, 2017). After normalisation, the samples were integrated to remove batch effects following the standard Seurat 3 workflow (Stuart et al., 2019), using 3000 features and 60 components. Following integration, the standard Seurat 3 clustering workflow was carried out: The RunPCA() function was used to compute 60 principal components (PCs), the FindNeighbors() and FindClusters() commands were used with default parameters to identify cell clusters, and the FindMarkers() command was used with the MAST method from the MAST R package(Finak et al., 2015) to identify differentially expressed genes. Cells were mapped to 2-dimensional UMAP coordinates using the RunUMAP() function with default parameters. Having used the Seurat 3 integration workflow to identify cell clusters in the presence of significant sample batch effects, we decided to return to the SCTransform normalised counts and use the ComBat() batch-correction technique from the sva R package for all other downstream analysis(Johnson et al., 2007; Leek and Storey, 2007). The reasons for doing so were (1) the need to include all genes (only a subset are used in the Seurat 3 integration workflow) (2) the greater mathematical interpretability of ComBat() and reduced risk of overfitting and (3) the desire for interpretable, non-negative units of gene expression. Using ComBat(), we corrected both for differences in mean and scale of expression. To avoid the introduction of artificial co-expression patterns in subsequent analyses, all values which were zero in the SCTransform-normalised counts matrix were reset to zero in the batch corrected matrix as well. All code used in the analysis is available at the GitHub development platform (GitHub code repository: https://github.com/perslab/fred-2022).

Single-cell RNA sequencing cell cluster annotations

To annotate the cell clusters, we drew on existing scRNA-seq studies of the human liver (Aizarani et al., 2019; MacParland et al., 2018). First we used the Seurat label reference-based label transfer workflow (Stuart et al., 2019) to match annotations from MacParland et al. (MacParland et al., 2018) to our cells. Second, to match our clusters to Aizarani et al. (Aizarani et al., 2019), in the absence of published cell annotations we used Spearman's ρ correlations between cluster marker genes to match our cell clusters to theirs. We found that there was generally a good correspondence between the cell annotations predicted by the two comparisons, allowing us to label most of our clusters with high certainty. The cell clusters for which the comparisons did not produce a clear result were annotated manually based on marker genes. Cell cluster gene expression panels were made using the ggplot2 R

package (Wickham, 2016), and heatmaps were made with the pheatmap R package (CRAN - Package pheatmap).

Bulk RNA-seq data processing

RNA-seq fastq files were downloaded from the European Nucleotide Archive (study (Sequence) PRJNA512027) using the ENA browser tools (Gerhard et al., 2018; GitHub - enasequence/enaBrowserTools). Sequence quality was assessed using fastqc (version 0.11.5) and MultiQC (version 1.8), and samples with fewer than 200m sequences were removed (Babraham Bioinformatics). Sequences were aligned to the GRCh38 (Ensembl 93) genome assembly using STAR (version 2.7.3a) (Babraham Bioinformatics), with sjdbOverhang set to 82. Binary Alignment Map (BAM) files were sorted using samtools sort (version 1.9) and reads were counted using htseq-count (version 0.11.2). The raw gene counts were assembled as a matrix and normalised using the DESeq data set FromMatrix(), estimateSizeFactors() and counts() functions from the DESeq2 R package(Babraham Bioinformatics). Several samples (DLDR_0037-46 and DLDR_61-62) were relabelled as fibrotic on advice of the corresponding author responsible for generating the data set and an outlier sample (DLDR_0181) was omitted after visual inspection of Principal Component plots. The data set comprised a total of 160 obese cases and 24 controls labelled by clinical status.

Robust Weighted Gene Co-expression Network Analysis

Cell type subsetting and gene filtering

We divided the normalised, batch-corrected counts by coarse cell type to capture cell type specific networks while ensuring sufficient heterogeneity and cell numbers to detect biological signal. Genes expressed in fewer than 20 cells in a cluster were removed. Principal component analysis was carried out using the RunPCA function to compute 100 Principal Components (PCs). Genes were then ranked by their highest absolute loading value on any given PC and the top 5,000 genes within each cell cluster were selected for co-expression analysis. In what follows, unless otherwise indicated, *P*-values were adjusted for multiple testing using the Benjamini-Hochberg method (Benjamini and Hochberg, 1995).

rWGCNA adjacency and Topological Overlap Matrix computation

Robust Weighted Gene Co-expression Analysis was carried out separately within single-cell and bulk RNA-seq data using the WGCNA R package (version 1.68) as described in Pfisterer et al. (Pfisterer et al., 2020), resulting in 301 and 14 modules detected within the single-cell and bulk data, respectively. The main outputs of the analysis were lists of the genes assigned to each module, along with their gene intramodular connectivity ('kIM') scores, representing a continuous measure of a gene's centrality in a module (Langfelder and Horvath, 2008).

Post-rWGCNA gene module filtering

Of the original 301 single-cell rWGCNA modules detected, we excluded 48 modules from cells identified as erythrocytes. Next, we computed separate module activity scores in our single-cell data, the MacParland data set and the Gerhard data set, by scaling by the standard deviation (but not centering) the normalised expression of each gene, normalising each module's kIM weights to sum to 1, and using these scaled kIMs as weights in a weighted average of gene expression in each module. These scores were finally scaled by their standard deviation across samples (hereafter 'module activity scores'). We excluded modules whose activity score in our single-cell data set was more correlated (Pearson's ρ) with a cell's number of RNA molecules, proportion of mitochondrial RNA, or donor origin than with biological cluster membership, leaving 202 modules. Next, we filtered out modules with a large fraction of co-expressed genes from ambient RNA as follows. The CELLEX toolkit (Timshel et al., 2020) was used on the normalised single-cell data to compute gene specificity scores (0-1) for each cell cluster. For each module and cell cluster, we computed as a test statistic the dot product of the gene module kIM scores with the matching gene CELLEX specificity scores. A null distribution was obtained for each statistic by recomputing 9999 replicates using randomly sampled CELLEX scores from the cell cluster, resulting in one-sided percentile *P*-values. *P*-values were corrected for multiple testing (Benjamini-Hochberg, n=2424). Gene modules with a significant enrichment for another cell cluster and no significant enrichment for the cell cluster of origin were discarded, leaving 147 modules. Next, to ensure that single-cell gene modules were not specific to our data set, we used the WGCNA modulePreservation() function to assess whether co-expression of single-cell gene modules was preserved in the Gerhard *et al.* bulk RNA-seq data set. After adjusting *P*-values for multiple testing (Benjamini-Hochberg, n=147), 22 single-cell modules were retained for testing for association with NASH in the bulk RNA-seq samples.

Module preservation in control single-cell RNA-seq data set

We tested the extent to which the co-expression of the remaining 22 gene modules was preserved in the matching cell types of the MacParland et al. single-cell data set (**Fig. 2C**) using the same method as for the bulk data set described above. Modules from cell types without a match in the MacParland et al. data set (Dendritic cells and Hepatic stem cells) were omitted in this step.

Module association with disease condition

In order to identify modules associated with NASH, we carried out linear regression analyses using module activity scores in the bulk RNA-seq data set. We extracted subsets of the bulk data and converted categorical labels to scores of 0, 1 or 2 for a given condition, with "normal" samples (n=24) encoded as 0. For steatosis, samples rated as "steatosis 2" (n=34) or "steatosis 3" (n=16) were included and converted to 1 and 2; for lobular inflammation, we included samples rated as "lob inflam 1" (n=38) or "lob inflam 2" (n=13) and encoded them as 1 and 2, respectively; for fibrosis, samples labelled "fibrosis 3" (n=19) were included, encoded as 1. When contrasting lobular inflammation with fibrosis, "lob inflam 1" and "lob inflam 2" samples were contrasted with those labelled "fibrosis 3".

Module activity scores in the bulk RNA-seq data set were generated in the same way as for the single-cell data, as described above. Using both bulk and single-cell modules, we computed a multivariate linear regression model with module activity as the dependent variable and steatosis, lobular inflammation or fibrosis in turn as the "condition" regressor:

$$\text{module expression} \sim \text{condition} + \text{sex} + \text{age} + \text{BMI} + \varepsilon$$

i.e. predicting module activity from condition, while adjusting for sex (binary), age (continuous) and BMI at time of surgery (continuous) of the bulk RNA-seq sample donor, with $\varepsilon \sim N(0, \sigma^2)$. In order to obtain non-parametric confidence intervals and *P*-values, we used the R boot package's boot() command to generate R=999 case-bootstrapped estimates of the regression coefficients. Two-sided percentile *P*-values were computed as the proportion of bootstrap coefficient estimates with the opposite sign of the original coefficient estimate, i.e.

$$\frac{\Sigma_i^R 1_{B_i \leq 0}}{R} \text{ if } B > 0\text{, and } \frac{\Sigma_i^R 1_{B_i \geq 0}}{R} \text{ otherwise}$$

where B denotes the original coefficient estimate, B_i the bootstrapped coefficient estimates and 1_x denotes the function that evaluates to 1 if x is true and 0 otherwise.

Bootstrap percentile p-values were adjusted for multiple testing within each condition (n=22 modules) and a percentile cut-off corresponding to a two-sided hypothesis test (P<0.025) was used to obtain lists of disease-associated modules (Sun et al., 2006).

Functional module annotation through gene set enrichment testing

To obtain functional annotations for the disease-associated gene modules we queried the Gene Ontology Biological Process, Molecular Function and Cellular Component databases (GO BP, MF and CC) (Ashburner et al., 2000; Carbon et al., 2019), the Kyoto Encyclopaedia of Genes and Genomes (KEGG) (Kanehisa and Goto, 2000) and Reactome (Jassal et al., 2020). To address overlap and the resulting statistical dependence between gene sets, we measured enrichment test using the regression_selected_pathways() command from the Gene Enrichment Gene-set Enrichment with Regularized Regression (GERR) R package (Fang et al., 2019). The method treats module genes as outcome and gene sets as coefficient vectors in a linear regression with elastic net regularization. It returns the subset of gene sets associated with modules along with *P*-values computed using Fisher's test, which we adjusted for the total number of tests across three databases (Benjamini-Hochberg, n=528,804). For plotting, we used REVIGO with default parameters to remove redundant GO terms (Supek et al., 2011).

Mapping of candidate genes for genome-wide association with NAFLD to modules

The 215 potentially relevant genes for NAFLD (diagnosed using elevated ALT as a proxy for NAFLD) recently presented by Vujkovic et al. (Vujkovic et al., 2021), were mapped to our modules using hypergeometric enrichment tests. The genes are the predicted effector genes for 77 genome-wide loci significantly associated with NAFLD. In brief, the candidate genes are based on DEPICT gene prediction, coding variant linkage analysis, QTL analysis, and annotation enrichment, and PPI networks.

Supplementary results

Mapping of bulk modules to single-cell data set

Given that the 22 cell type-specific modules were derived from our single-cell data set, which comprissed a limited number of NASH-patients, we next derived modules based on the Gerhard et al. study comprising of bulk RNA-seq data from 184 liver donors with phenotypes ranging from normal, through steatosis and inflammation to severe fibrosis. Performing rWGCNA, we identified 14 robust modules (M-Ger-1 to 14). Among those, four modules associated with inflammation and six with fibrosis (Suppl. **Fig. S3A**). We next calculated the activity of these modules across the different cell types in our single-cell data set and found that although most modules were expressed throughout the liver cell types, some of the bulk modules mapped to specific cell populations (Suppl. **Fig. S3B**). Notably, M-Ger-4, which mapped exclusively to dendritic cells, while M-Ger-13 was highly specific to plasma cells, despite these constituting relatively rare cell populations in the liver. (No module was specific for hepatocytes.) In summary, we can show that even though hepatocytes make up the largest fraction of cells in the liver, analyses of bulk RNA-seq data will still be comprised of gene expression from multiple cell types.

Supplementary figures

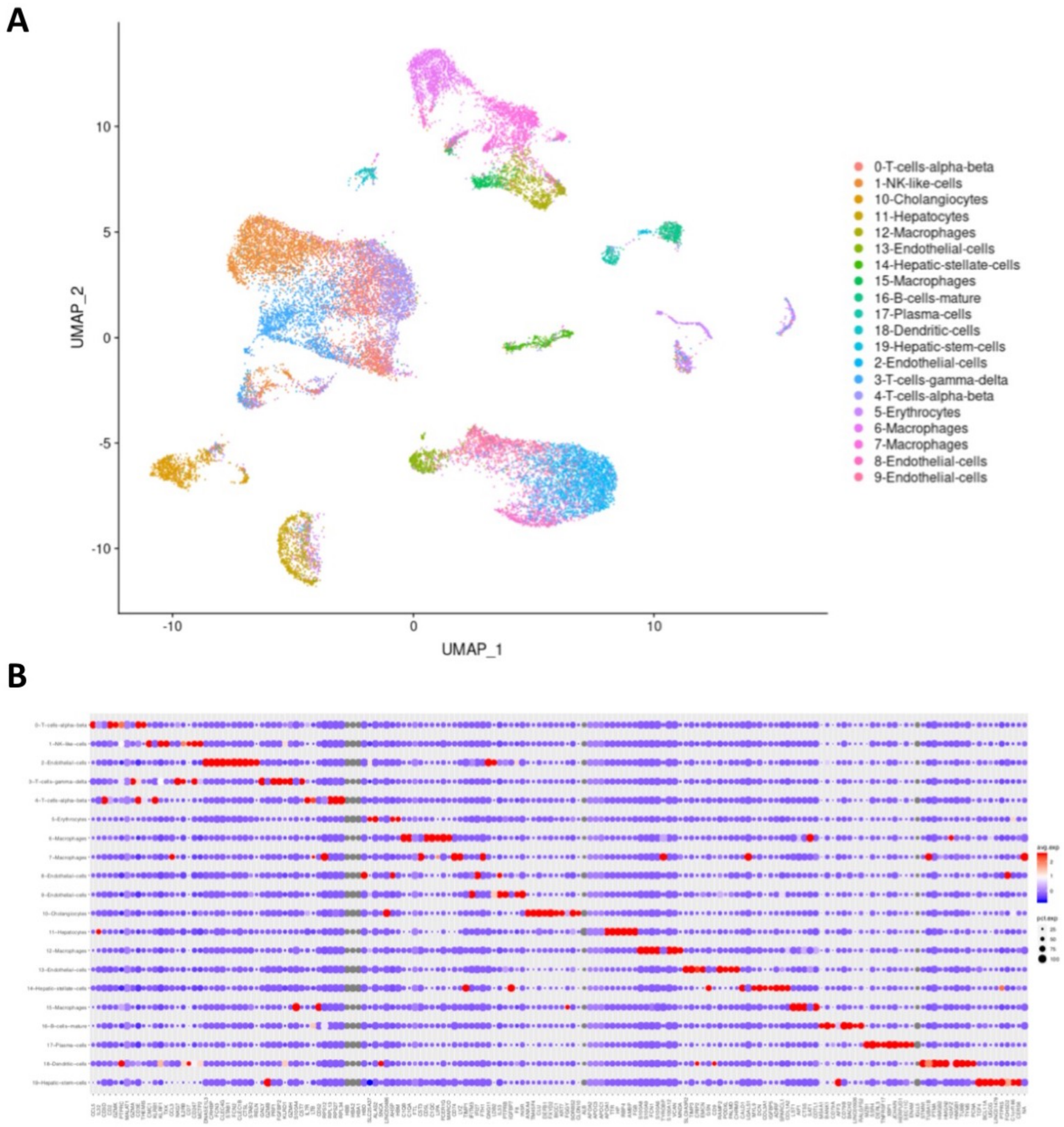

Figure S1. Automated clustering and transfer of labels generates a cell type specific data set. (A) UMAP showing the original clusters with the labels transferred from the MacParland and the Aizarani data sets. (B) Dot plot showing the expression of the top 10 differentially expressed cluster markers, indicating both expression level and fraction of cells in which the gene is expressed.

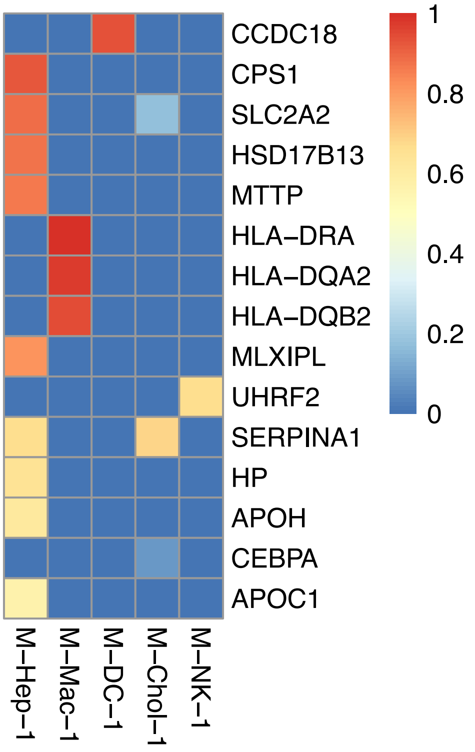

Figure S2. NAFLD associated gene expression in single-cell modules. Based on 77 NAFLD associated loci, 214 predicted genes were tested for presence and centrality. Only genes expressed in our modules are shown. Three modules were significantly enriched for genes associated with significant loci for NAFLD, M-Hep-1 (P=1.65x10^{-7}), M-Chol-1 (P=5.05 x10^{-5}) and M-Mac-1 (P=5.05 x10^{-5})

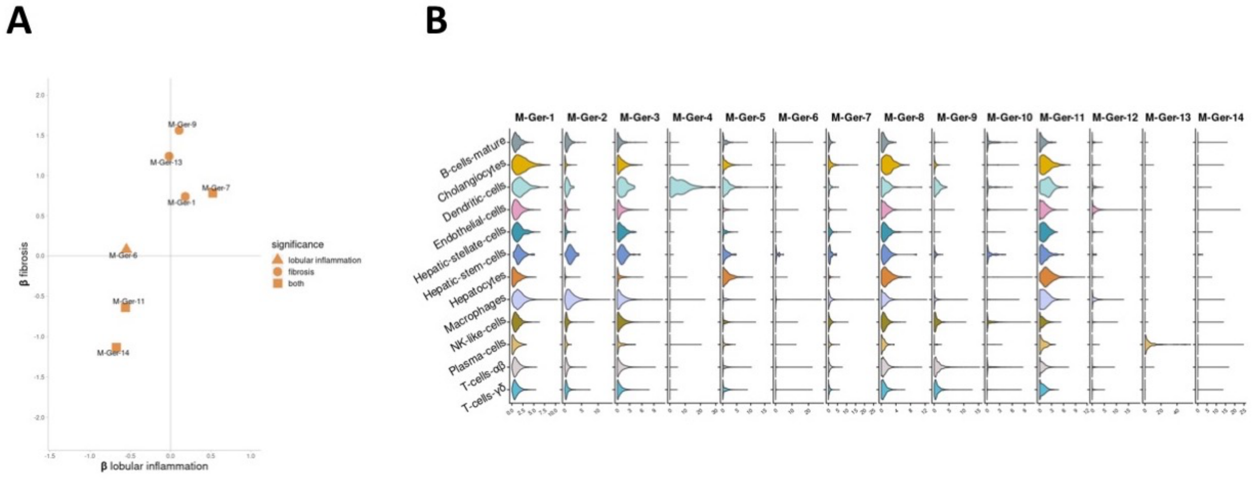

Figure S3. WGCNA reveals cell type specificity of bulk modules. (A) WGCNA modules from the Gerhard et al. bulk data set associated with inflammation and fibrosis. The vertical and horizontal axes correspond to standard deviation differences in module activity for each unit change in lobular inflammation (encoded as 0, 1 or 2) or fibrosis (0 or 1). The plot includes only modules with statistically significant association to at least one condition using bootstrap percentile P-values corrected for multiple testing within each phenotype (Benjamini-

Hochberg). (B) Violin plot of bulk RNA-seq module activity, a weighted mean of normalized gene expression, within cell clusters.

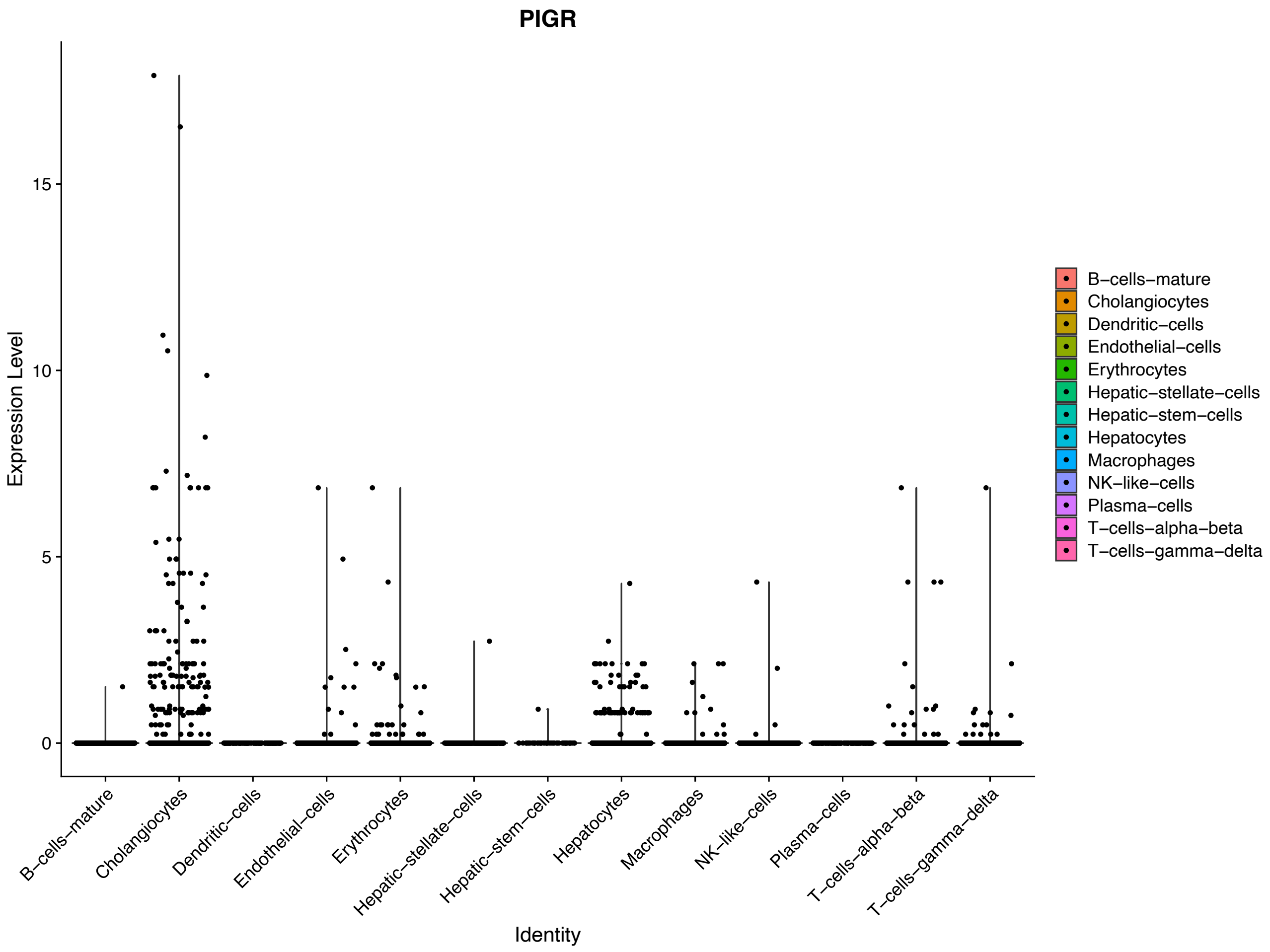

Figure S4. Expression of *PIGR* in human liver.

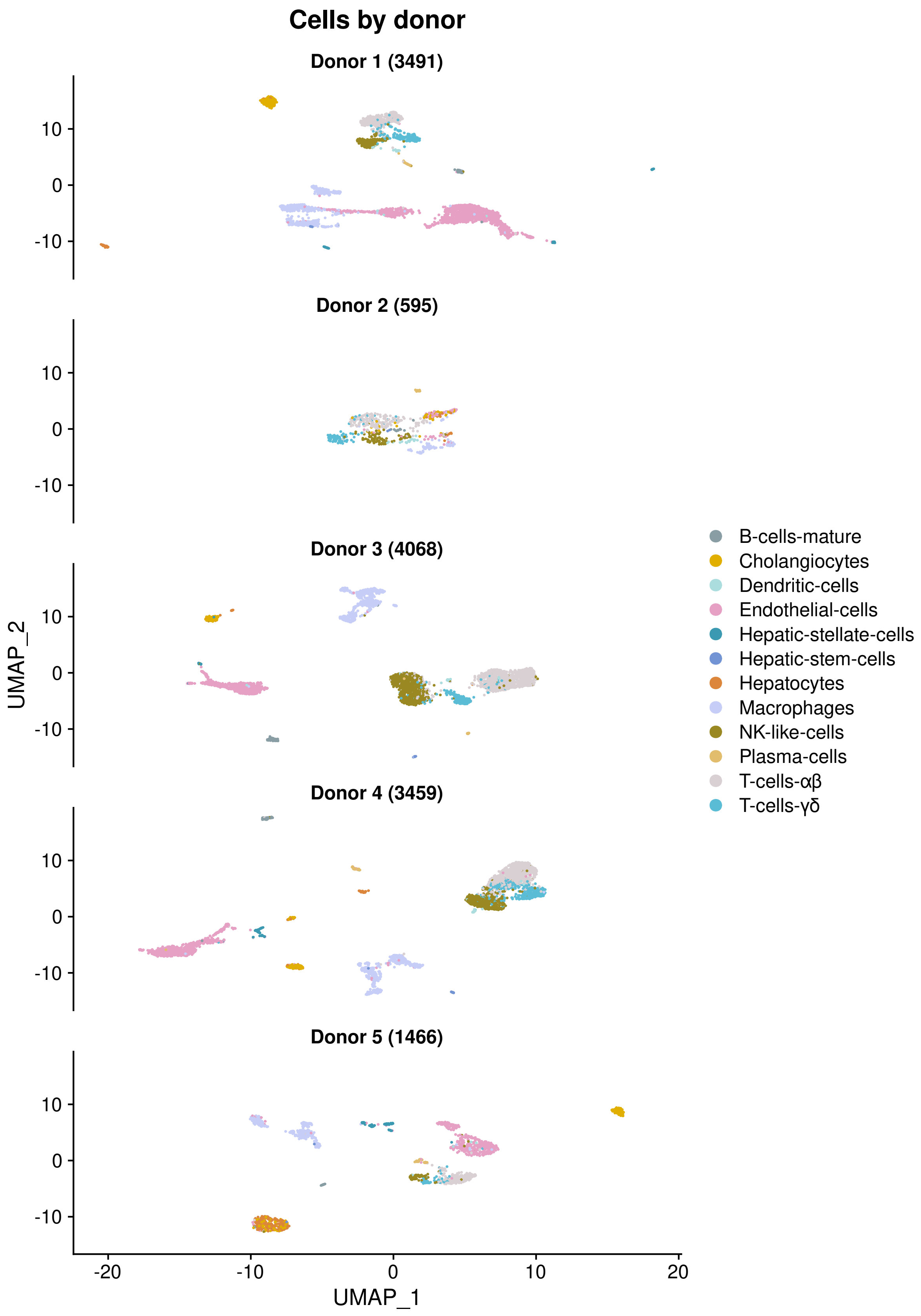
Cells by donor
Donor 1 (3491)
Donor 2 (595)
Donor 3 (4068)
Donor 4 (3459)
Donor 5 (1466)
10
0
-10
-20
20
UMAP_1
UMAP_2
B-cells-mature
Cholangiocytes
Dendritic-cells
Endothelial-cells
Hepatic-stellate-cells
Hepatic-stem-cells
Hepatocytes
Macrophages
NK-like-cells
Plasma-cells
T-cells-αβ
T-cells-γδ

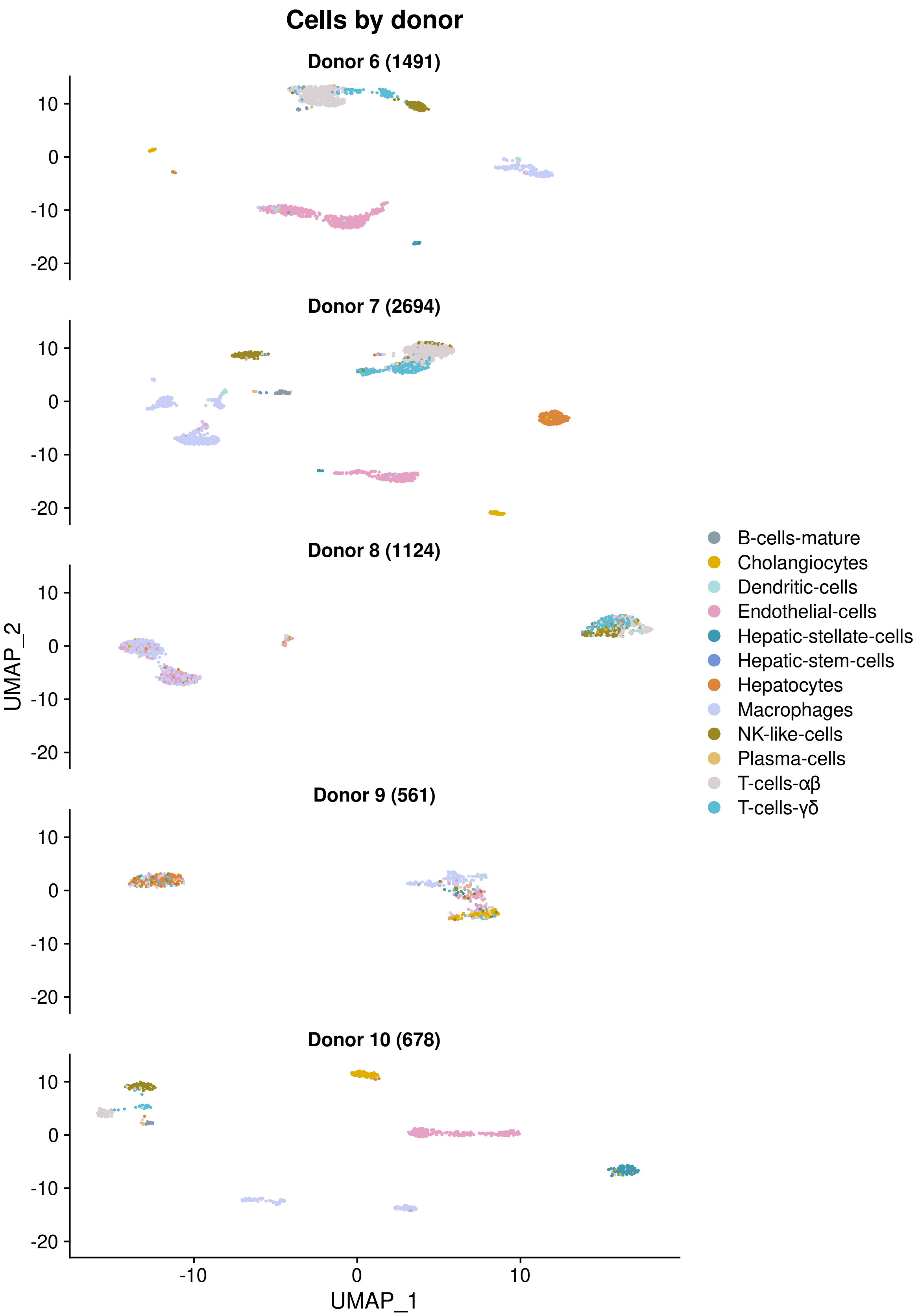
Cells by donor
Donor 6 (1491)
Donor 7 (2694)
Donor 8 (1124)
Donor 9 (561)
Donor 10 (678)
UMAP_2
UMAP_1
10
0
-10
-20
-10
0
10
B-cells-mature
Cholangiocytes
Dendritic-cells
Endothelial-cells
Hepatic-stellate-cells
Hepatic-stem-cells
Hepatocytes
Macrophages
NK-like-cells
Plasma-cells
T-cells-αβ
T-cells-γδ

Figure S5. Individual UMAP projection of the 10 donors. Number of cells by donor after quality control, after normalizing, running PCA and performing UMAP on each sample separately.

Supplementary references

10x Genomics Creating a Reference Package with cellranger mkref -Software -Single Cell Gene Expression -Official 10x Genomics Support.

Aizarani, N., Saviano, A., Sagar, Mailly, L., Durand, S., Herman, J.S., Pessaux, P., Baumert, T.F., and Grün, D. (2019). A human liver cell atlas reveals heterogeneity and epithelial progenitors. Nature *572*, 199–204.

Ashburner, M., Ball, C.A., Blake, J.A., Botstein, D., Butler, H., Cherry, J.M., Davis, A.P., Dolinski, K., Dwight, S.S., Eppig, J.T., et al. (2000). Gene ontology: Tool for the unification of biology. Nat. Genet. *25*, 25–29.

Babraham Bioinformatics Babraham Bioinformatics - FastQC A Quality Control tool for High Throughput Sequence Data.

Bedossa, P., Poitou, C., Veyrie, N., Bouillot, J.L., Basdevant, A., Paradis, V., Tordjman, J., and Clement, K. (2012). Histopathological algorithm and scoring system for evaluation of liver lesions in morbidly obese patients. Hepatology *56*, 1751–1759.

Benjamini, Y., and Hochberg, Y. (1995). Controlling the False Discovery Rate: A Practical and Powerful Approach to Multiple Testing. J. R. Stat. Soc. Ser. B *57*, 289–300.

Carbon, S., Douglass, E., Dunn, N., Good, B., Harris, N.L., Lewis, S.E., Mungall, C.J., Basu, S., Chisholm, R.L., Dodson, R.J., et al. (2019). The Gene Ontology Resource: 20 years and still GOing strong. Nucleic Acids Res. *47*, D330–D338.

CRAN - Package pheatmap CRAN - Package pheatmap.

Dixon, J.B., Bhathal, P.S., Hughes, N.R., and O'Brien, P.E. (2004). Nonalcoholic fatty liver disease: Improvement in liver histological analysis with weight loss. Hepatology *39*, 1647–1654.

Fang, T., Davydov, I., Marbach, D., and Zhang, J.D. (2019). Gene-set Enrichment with Regularized Regression. BioRxiv 659920.

Finak, G., McDavid, A., Yajima, M., Deng, J., Gersuk, V., Shalek, A.K., Slichter, C.K., Miller,

H.W., McElrath, M.J., Prlic, M., et al. (2015). MAST: A flexible statistical framework for assessing transcriptional changes and characterizing heterogeneity in single-cell RNA sequencing data. Genome Biol. *16*.

Gerhard, G.S., Legendre, C., Still, C.D., Chu, X., Petrick, A., and DiStefano, J.K. (2018). Transcriptomic profiling of obesity-related nonalcoholic steatohepatitis reveals a core set of fibrosis-specific genes. J. Endocr. Soc. *2*, 710–726.

GitHub - enasequence/enaBrowserTools GitHub - enasequence/enaBrowserTools: A collection of scripts to assist in the retrieval of data from the ENA Browser.

Jassal, B., Matthews, L., Viteri, G., Gong, C., Lorente, P., Fabregat, A., Sidiropoulos, K., Cook, J., Gillespie, M., Haw, R., et al. (2020). The reactome pathway knowledgebase. Nucleic Acids Res. *48*, D498–D503.

Johnson, W.E., Li, C., and Rabinovic, A. (2007). Adjusting batch effects in microarray expression data using empirical Bayes methods. Biostatistics *8*, 118–127.

Kanehisa, M., and Goto, S. (2000). KEGG: Kyoto Encyclopedia of Genes and Genomes. Nucleic Acids Res. *28*, 27–30.

Langfelder, P., and Horvath, S. (2008). WGCNA: An R package for weighted correlation network analysis. BMC Bioinformatics *9*, 559.

Leek, J.T., and Storey, J.D. (2007). Capturing Heterogeneity in Gene Expression Studies by Surrogate Variable Analysis. PLoS Genet. *3*, e161.

MacParland, S.A., Liu, J.C., Ma, X.Z., Innes, B.T., Bartczak, A.M., Gage, B.K., Manuel, J., Khuu, N., Echeverri, J., Linares, I., et al. (2018). Single cell RNA sequencing of human liver reveals distinct intrahepatic macrophage populations. Nat. Commun. *9*.

Pfisterer, U., Petukhov, V., Demharter, S., Meichsner, J., Thompson, J., Batiuk, M., Asenjo Martinez, A., Vasistha, N., Thakur, A., Mikkelsen, J., et al. (2020). Identification of epilepsy-associated neuronal subtypes and gene expression underlying epileptogenesis. Nat. Commun. *11*.

Picelli, S. (2017). Single-cell RNA-sequencing: The future of genome biology is now. RNA Biol. *14*, 637–650.

Stuart, T., Butler, A., Hoffman, P., Hafemeister, C., Papalexi, E., Mauck, W.M., Hao, Y., Stoeckius, M., Smibert, P., and Satija, R. (2019). Comprehensive Integration of Single-Cell Data. Cell *177*, 1888-1902.e21.

Sun, L., Craiu, R. V., Paterson, A.D., and Bull, S.B. (2006). Stratified false discovery control for

large-scale hypothesis testing with application to genome-wide association studies. Genet. Epidemiol. *30*, 519–530.

Supek, F., Bošnjak, M., Škunca, N., and Šmuc, T. (2011). REVIGO Summarizes and Visualizes Long Lists of Gene Ontology Terms. PLoS One *6*, e21800.

Timshel, P.N., Thompson, J.J., and Pers, T.H. (2020). Genetic mapping of etiologic brain cell types for obesity. Elife *9*, 1–45.

Vilar-Gomez, E., Martinez-Perez, Y., Calzadilla-Bertot, L., Torres-Gonzalez, A., Gra-Oramas, B., Gonzalez-Fabian, L., Friedman, S.L., Diago, M., and Romero-Gomez, M. (2015). Weight loss through lifestyle modification significantly reduces features of nonalcoholic steatohepatitis. Gastroenterology *149*, 367-378.e5.

Vujkovic, M., Ramdas, S., Lorenz, K.M., Schneider, C. V, Lee, K.M., Serper, M., Carr, R.M., Kaplan, D.E., Haas, M.E., MacLean, M.T., et al. (2021). A genome-wide association study for nonalcoholic fatty liver disease 1 identifies novel genetic loci and trait-relevant candidate genes in the 2 Million Veteran Program. 3. Themistocles L. 12 Assimes *36*, 49.

Wickham, H. (2016). ggplot2: Elegant Graphics for Data Analysis (Springer-Verlag New York).
